# Multilevel analysis of trends and predictors of concurrent wasting and stunting among children 6–59 months in Ethiopia from 2000 to 2019

**DOI:** 10.3389/fnut.2023.1073200

**Published:** 2023-09-01

**Authors:** Aklilu Abrham Roba, Öznur Başdaş

**Affiliations:** ^1^College of Health and Medical Sciences, Haramaya University, Dire Dawa, Ethiopia; ^2^Faculty of Health Sciences, Erciyes University, Kayseri, Türkiye

**Keywords:** Trends, predictors, concurrent wasting and stunting, multi-level analysis, DHS

## Abstract

**Introduction:**

Emerging evidence indicates that children can be concurrently wasted and stunted (WaSt), increasing their mortality risk. However, more is needed to know about WaSt in Ethiopia. Therefore, this study aimed to determine the trends and predictors of WaSt using Ethiopian Demographic and Health Survey datasets from 2000 and 2019.

**Methods:**

The study included a total weighted sample of 34,930 children aged 6–59 months. Descriptive and weighted multilevel mixed-effects (fixed and random effects) logistic regression analyses were carried out. The Intraclass Correlation Coefficient (ICC) and the Median Odds Ratio (MOR) were calculated.

**Results:**

The prevalence of WaSt was 1,682 (4.82%) with a significantly decreasing trend, yielding a percent change of −57.51% (−69.37% to −23.52%) from 2000 to 2019. In the adjusted model, the odds of WaSt increased in boys, children with a shorter preceding birth interval, small birth size, delayed initiation of complementary foods, diarrhea, fever, and anemia, mother’s lack of formal education, and being a farmer, and poor/middle wealth index, and lack of mass media exposure. WaSt was inversely related to the child’s age. Adjusted ICC and MOR were 31.16% and 3.20%, respectively.

**Conclusion and recommendations:**

The study highlights the importance of considering individual and community-level factors to address WaSt, such as timely initiation of complementary foods, improving access to health services, quality diet, and prevention of communicable diseases. Furthermore, programs that have positive impacts on formal education and employment opportunities for girls, as well as that increase access to mass media, are required.

## Introduction

Stunting (linear growth faltering) is chronic undernutrition that prevents children from reaching their physical and cognitive potential (1, 2). On the other hand, wasting (ponderal growth faltering) is a devastating emergency associated with higher mortality risk if not adequately treated (1). Globally, stunting and wasting affected 149.2 million and 45.4 million under-five children, respectively (3). Concurrent wasting and stunting (*z* scores lower than −2 in both weight for height and height for age) is the co-occurrence of wasting and stunting in a child (4). Until recently, it was believed that wasting and stunting occur independently in a child, and the interaction between them was not considered of public health importance (5). This hypothesis is mainly based on age: stunting increases with age but wasting decreases (4, 6). However, emerging evidence shows a child can have concurrent wasting and stunting (WaSt) (4, 5, 7–9).

The prevalence of WaSt varies according to the burden of wasting and stunting and geographic locations. For example, it was 5% in Karamoja, Uganda (8), 6.2% in Niakhar, Senegal (4), less than 3.5% in Mozambique (5), 5.8% in Kersa, Ethiopia (7), and 5% of Bangladeshi, Indian, and Pakistani children (10). A meta-analysis in 2017 showed that the pooled prevalence of WaSt in 84 countries was 3.0%, ranging from 0 to 8.0% (11). The exact cause of WaSt was not clearly determined. Still, it may share the underlying and fundamental causal factors of wasting and stunting, such as infectious diseases, environmental enteric dysfunction, a diet with inadequate nutrients, and suboptimal infant feeding and caring practices (12, 13). In the body of literature, male sex, child age, cough/ acute respiratory infection, diarrhea, malaria/fever in the last 2 weeks before the survey, multiparity, not treating drinking water at the point of use, conflict, and instability were found to be predictors of WaSt (7, 8, 14).

In the national and international guidelines that were prepared to manage malnutrition, there is no case definition for WaSt (15). Thus, children with WaSt get less attention in the health system and are treated as wasted children. However, they are not only wasted but also stunted, and the interaction between wasting and stunting makes them susceptible to poor health outcomes. As the severe acute malnutrition definition (presence of bilateral pitting edema or severe wasting) does not account for the stunting status of the child, the national and WHO guidelines have no room to identify those children at the highest risk of dying, prompt management, and monitoring either at the community or health institutions level (15). Although WaSt children had the highest mortality risk, they were neglected in the health system due to a lack of case definition and appropriate management protocol (9).

Globally, the Demographic and Health Surveillance (DHS) program was introduced by the United States Agency for International Development (USAID) in 1984 (16). However, in Ethiopia, the first survey was conducted in 2000 by the Central Statistical Agency (17). The surveys have generated nationally representative data on different topics, including demographics, maternal and child health, and nutrition, that provide estimates at the national and regional levels and for urban and rural areas (18). The DHS data was hierarchical or multilevel in structure, i.e., children were nested in the households, and households were nested in the enumeration areas (clusters). Households living in the same clusters may resemble each other more than those households from different clusters as they share similar multiple determinants of health and nutrition (19). Thus, the multilevel logistic regression analysis was carried out to identify predictors of WaSt because it incorporates cluster-specific random effects and leads to more accurate inferential decisions for hierarchical data (19–21).

Several studies have been conducted regarding wasting, stunting, and underweight in Ethiopia (22–28). However, little is known about the prevalence and predictors of WaSt. Thus, with a multi-level analysis approach, this study assessed the trends and predictors of WaSt among children 6–59 months using DHS data from 2000 to 2019.

## Methods and materials

### Data

The data for this study were drawn from nationally representative Ethiopian DHS data conducted in 2000, 2005, 2011, 2016, and 2019. DHS is a nationally representative household survey with a cross-sectional design. Data were collected on various topics, including child health and nutrition (18). It was retrieved from the (DHS) program’s official database website.^1^ The DHS is conducted every 5 years since 2000 and follows the standard sampling procedure, data collection, and coding (16). The children’s files were used for this study. The Demographic and Health Surveys (DHS) Program data archivist granted permission to use the data for the present study.

The DHS uses two-stage stratified sampling to recruit participants during the surveys. Regions/provinces are stratified into urban and rural areas at stage one. Enumeration areas or clusters from each region are sampled proportional to size. Then, a list of households within the selected EAs was generated as a sampling frame. Later in the second stage, a fixed number of households per cluster was selected from the sampling frame (18).

### Inclusion and exclusion criteria

**Figure 1:**
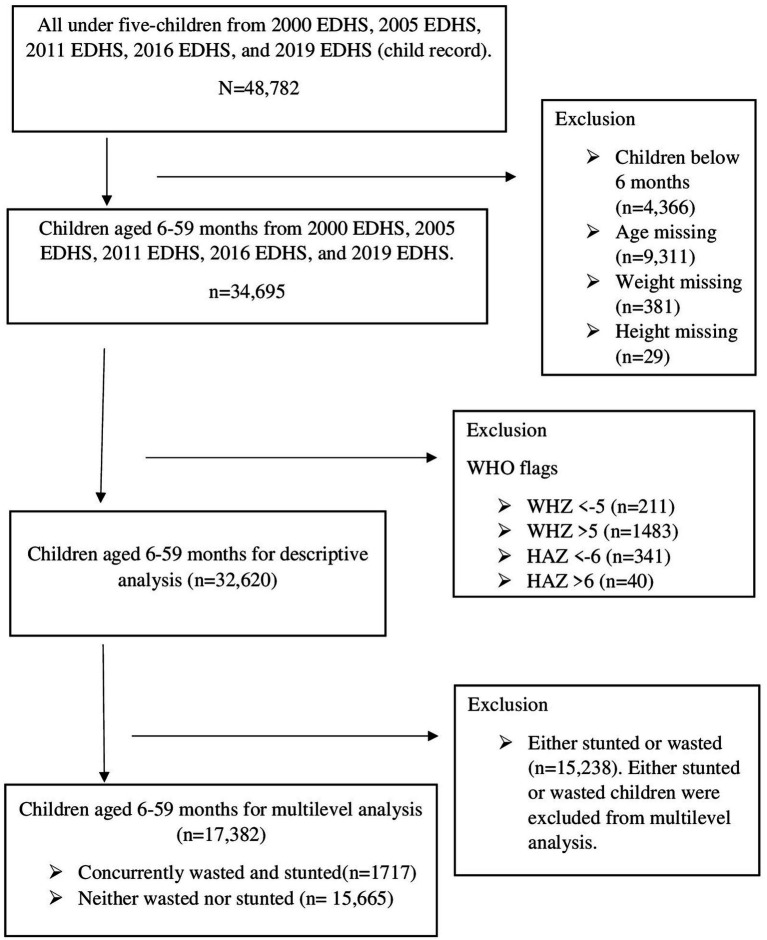
Flow chart for inclusion and exclusion criteria; data from EDHS 2000–2019.

A total of 34,930 weighted samples (32,620 unweighted samples) were included in the descriptive study. Neither wasted nor stunted children comprise 16,178 (46.31%), while 1,682 (4.82%) were concurrently wasted and stunted. For the multi-level analysis, we included 17,859 weighted samples (17,382 unweighted samples) (Figure 1).

### Outcome and explanatory variables

The outcome variable of the study was concurrent wasting and stunting (WaSt). WaSt is defined as a child being wasted and stunted simultaneously (14). The explanatory variables were categorized as individual-level (level-1) and community-level (level-2) factors. The selected child-level and household variables were based on previous studies (4, 5, 7, 8, 11). Child sex (male, female), age in months (6–11, 12–23, 24–35, 36–47, and 48–59 months), preceding birth interval (less than 2 years, 2 years, 3 years, and 4 years and above), birth size (small/very small, average/large), timely initiation of complementary foods (yes/no), received vitamin A in the last 6 months (yes/no), anemia (yes/no), diarrhea, cough, or fever in the 2 weeks before the survey (yes/no) were considered as child-level variables. Education status of the mother (no formal education, have formal education), occupation of the mother (not working, agriculture, business, and others), wealth status (poor, middle, rich), and women decide on health, purchases, and visits either alone or jointly with the partner (yes/no) were considered as household-level variables. In this analysis, individual-level characteristics (level-1) represent the summation of both child-level and household-level variables. Community-level (level-2) variables were residence (urban/rural), round time to get drinking water (water on premises, 30 min or less, more than 30 min), community media exposure (media exposure: newspaper, radio, TV at least once a week), type of toilet (improved, unimproved, or open defecation). The type of toilet is included under the community-level variable because some households use shared toilet facilities. Similarly, as the type of information disseminated may vary from cluster to cluster due to contextual, language, and regional variations, access to mass media was included under the community-level variable.

### Statistical analyses

Data extraction, recoding, and analysis were carried out using STATA version 14 software. Weighted frequencies and percentages were computed for all variables using sampling weight (v005/1,000,000) to account for unequal probability of selection given different population sizes within enumeration areas/ clusters. Multicollinearity between variables was tested using the variance inflation factor (VIF), included those with a VIF less than 5 (19, 29). The cluster number (v001) was considered as a unit of analysis for the community-level factors. Variables with a *p*-value less than 0.05 in bivariate analysis were selected for multivariable analysis. The following four weighted multilevel logistic regression models: the empty model (with no independent variables), model II (adjusted for individual-level variables only), model III (adjusted for community-level variables only), and model IV (adjusted for both individual-level and community-level variables simultaneously) were fitted. Finally, an adjusted odds ratio (AOR) with a 95% confidence interval (CI) was reported. The Akaike information criterion (AIC), the Bayesian information criterion (BIC), and the log-likelihood ratio test were used to estimate the goodness of fit of the models, where the model with the highest value of the Log-likelihood test and with the lowest values of AIC and BIC was considered to be the best-fit model (21).

Variability across the clusters was measured by the likelihood ratio, Intraclass Correlation Coefficient (ICC), Median Odds Ratio (MOR), and Proportional Change in Variance (PCV). Children living in the same cluster may be more similar than those children living in other clusters as they share similar environments, public health facilities, and other area characteristics (19). The ICC represents the proportion of the total observed individual variation in the concurrent wasting and stunting attributable to between-cluster variation. If the ICC was close to 0, children with concurrent wasting and stunting from the same cluster were no more different from a random sample of children from the general population. On the other hand, if the ICC were close to 1, all children in the same cluster would have the same outcome (30).

The MOR measures how much variability in the WaSt exists between the clusters by comparing two children from two randomly selected different clusters, that is, the odds of WaSt between the child at the cluster with a higher risk of WaSt and the cluster with a lower risk of WaSt (31, 32).


$$\mathrm{MOR}=\exp.\left[\surd \left(2\times V_{A}\right)\times 0.6745\right]\approx \exp.\left(0.95\surd V_{A}\right).$$

The PCV measures the total variation in concurrent wasting and stunting attributed to individual and community-level factors in the multilevel model (31).


$$\mathrm{PCV}=\left(V_{a}-V_{b}\right)/V_{a}{}^{*}100.$$

Where V_a_ is the variance in the initial model and V_b_ is the variance of the model with more terms (31).

## Results

### Characterization of child-level variables

The median age of children was 32 months, with the 25th and 75th percentiles of 18 and 46 months. Among them, 17,795 (50.95%) were boys, 15,756 (55.13%) had a preceding birth interval of 2 years or less, and 8,749 (28.80%) had small/very small birth sizes. The prevalence of diarrhea, cough, and fever in the last 2 weeks before the survey was 17.64%, 24.18%, and 17.88%, respectively. Around three-fourths (74.56%) of children had timely initiation of complementary feeding (6–8 months). Only 10,113 (48.83%) of children were not anemic (Table 1).

**Table 1:** Sociodemographic, nutrition and other child-level characteristics in Ethiopia; data from EDHS 2000–2019.

Variables	Characteristics	Weighted sample	Weighted percentage
Nutritional status	Neither wasted nor stunted	16,178	46.31
	Either wasted or stunted	17,070	48.87
	Concurrently wasted and stunted	1,682	4.82
Sex	Boys	17,795	50.95
	Girls	17,135	49.05
Age in months	6–11	4,042	11.57
	12–23	7,587	21.72
	24–35	7,454	21.34
	36–47	8,122	23.25
	48–59	7,725	22.12
Birth order	1st	6,320	18.09
	2nd–3rd	10,773	30.84
	4th–6th	11,365	32.54
	7th and above	6,472	18.53
Preceding birth interval	Less than 2 years	5,808	20.32
	2 years	9,948	34.81
	3 years	6,462	22.61
	4 years and above	6,363	22.26
Birth size	Small/very small	8,749	28.80
	Average/large	21,627	71.20
Duration of breastfeeding	Not breastfed	854	2.45
	Less than 6 months	945	2.72
	6–12 months	5,006	14.39
	1–2 years	9,797	28.17
	More than 2 years	18,178	52.27
Timely initiation of complementary feeding	Yes	26,044	74.56
	No	8,886	25.44
Diarrhea in the last 2 weeks before the survey	Yes	5,372	17.64
	No	25,042	82.22
Cough in the last 2 weeks before the survey	Yes	7,365	24.18
	No	23,064	75.73
Fever in the last 2 weeks before the survey	Yes	6,245	17.88
	No	28,685	82.12
Received vitamin A in the last 6 months	Yes	15,258	47.04
	No	17,176	52.96
Anemia status	Severe	610	2.94
	Moderate	5,225	25.23
	Mild	4,764	23.00
	Not anemic	10,113	48.83

### Characterization of household-level variables

Females led around 4,637 (13.28%) households. Most mothers, 24,816 (71.04%), and more than half of the fathers, 16,249 (54.76%), had no formal education. Also, most mothers 14,580 (47.87%) were not working, while 23,441 (78.38%) fathers were farmers. 11,769 (45.41%) children were from poor households. Only 11,310 (34.56%) women reported that they could decide on health, purchases, and visits alone or jointly with their partner (Table 2).

**Table 2:** Sociodemographic and other household characteristics of parents in Ethiopia; data from EDHS 2000–2019.

Variables	Characteristics	Weighted sample	Weighted percentage
Sex of household head	Male	30,293	86.72
	Female	4,637	13.28
Marital status	Never married	160	0.46
	Married	32,070	91.81
	Living together	654	1.87
	Widowed	548	1.57
	Divorced	1,046	2.99
	Not living together	453	1.30
Religion	Orthodox Christian	14,089	40.56
	Muslim	12,207	35.14
	Protestant	7,495	21.58
	Others	947	2.73
Maternal educational status	No formal education	24,816	71.04
	Formal education	10,114	28.96
Maternal occupation	Not working	14,580	47.87
	Farmer/agriculture	7,573	24.86
	Business and others	8,303	27.26
Number of household members	Three or less	3,109	8.90
	Four to six	18,174	52.03
	Seven or more	13,647	39.07
Wealth status	Poor	11,769	45.41
	Middle	5,320	20.52
	Rich	8,831	34.07
Women empowered (decide on health, purchases, and visits alone or jointly with their partners)	No	21,414	65.44
	Yes	11,310	34.56

### Characterization of community-level variables

Most of the participants live in rural communities (87.33%), consume water from non-piped sources (84.59%), practice open defecation (53.85%), and have no access to newspaper, radio, or television at least once a week (86.03%) (Table 3).

**Table 3:** Community-level variables of WaSt in Ethiopia; data from EDHS 2000–2019.

Variables	Characteristics	Weighted sample	Weighted percentage
Residence	Urban	4,424	12.67
	Rural	30,506	87.33
Source of drinking water	Not piped	29,546	84.59
	Piped clean	5,383	15.41
Round trip time to obtain a water source	Water on premises	2,234	6.53
	30 min or less	20,177	58.98
	More than 30 min	11,800	34.49
Type of toilet	Open defecation	18,557	53.85
	Unimproved	11,730	34.04
	Improved	4,171	12.11
Access to newspaper, radio, or television at least once a week	Yes	4,880	13.97
	No	30,050	86.03

### Prevalence of concurrent wasting and stunting

**Figure 2:**
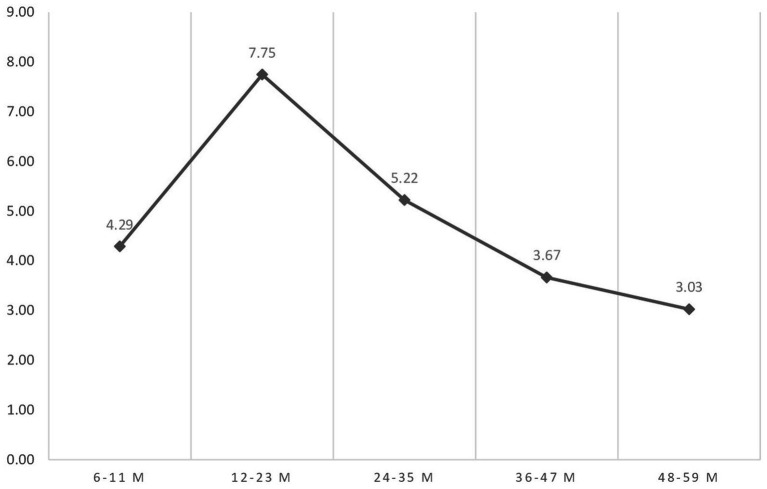
Prevalence of WaSt based on age of a child; data from EDHS 2000–2019.

The weighted prevalence of concurrent wasting and stunting was 7.39% in 2000 EDHS, 5.30% in 2005 EDHS, 4.52% in 2011 EDHS, 3.06% in 2016 EDHS, 3.14% in 2019 mini-EDHS, and the overall prevalence was 4.82% in Ethiopia. The prevalence of WaSt sharply increased from 6 to 11 months and peaked at 12 to 23 months. Then, it decreases slowly until 48–59 months (Figure 2).

### Trends of concurrent wasting and stunting

**Figure 3:**
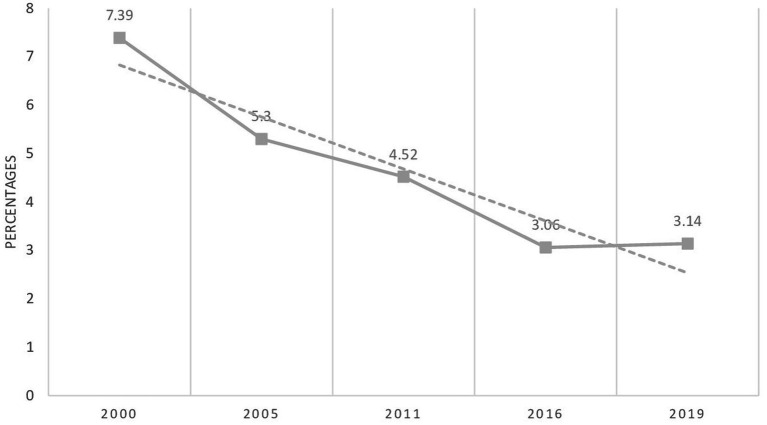
Trends in concurrent wasting and stunting in Ethiopia; data from EDHS 2000–2019.

**Figure 4:**
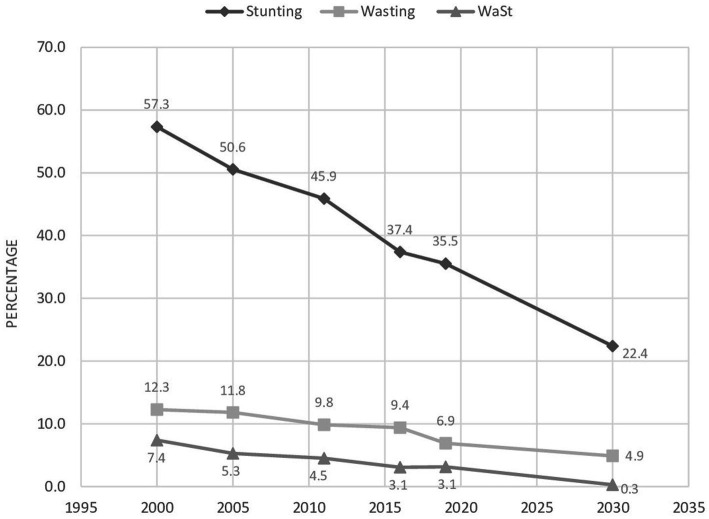
Trends and forecasting of stunting, wasting, and WaSt among children 6–59 months in Ethiopia; data from EDHS 2000–2019.

The trends of WaSt were declining, with a percent change of −57.51% between 2000 and 2019 in Ethiopia (Figure 3). Trends in stunting, wasting, and WaSt from 2000 to 2030 (forecasted for 2030) were presented in Figure 4.

### Predictors of concurrent wasting and stunting (fixed-effects)

The factors associated with WaSt in Ethiopia include child sex and age, preceding birth interval, birth size, time of initiation of complementary foods, diarrhea, fever, and anemia, education and occupation of the mother, wealth index, residence, time spent to obtain water, and media exposure. At the individual-level factors, the likelihood of WaSt was high among boys (aOR 1.81, 95% CI 1.49–2.20), children aged 12–23 months (aOR 4.56, 95% CI 3.20–6.50), 24–35 months (aOR 3.51, 95% CI 2.39–5.15), 36–47 months (aOR 2.44, 95% CI 1.65–3.61), and 48–59 months (aOR 1.85, 95% CI 1.22–2.80) compared to children 6–11 months. Shorter preceding birth intervals of less than 2 years (aOR 1.83, 95% CI 1.34–2.49) and 2 years (aOR 1.59, 95% CI 1.20–2.08) were more likely to be associated with concurrent wasting and stunting when compared to preceding birth interval of 4 years/more. Similarly, small/very small children were twice (aOR 2.51, 95% CI 2.05–3.08) higher odds of concurrent wasting and stunting than average/larger birth size children. Timely initiation of complementary foods in 6–8 months had 38% (aOR 0.62, 95% CI 0.49–0.79) lower odds of concurrent wasting and stunting than their counterparts. Children who had diarrhea (aOR 1.57, 95% CI 1.22–2.03) and fever (aOR 1.68, 95% CI 1.25–2.20) in the last two weeks before the survey had higher odds of concurrent wasting and stunting than their counterparts. Children with severe anemia (aOR 4.70, 95% CI 2.91–7.59), moderate anemia (aOR 2.51, 95% CI 1.96–3.21), and mild anemia (aOR 1.88, 95% CI 1.47–2.42) were higher odds of concurrent wasting and stunting than those with no anemia. Lack of formal maternal education (aOR 1.68, 95% CI 1.28–2.19) and being a farmer (aOR 1.39, 95% CI 1.01–1.92) were associated with concurrent wasting and stunting. Also, being from a poor household (aOR 1.98, 95% CI 1.47–2.68) and middle wealth index (aOR 1.70, 95% CI 1.23–2.33) had higher odds of concurrent wasting and stunting than wealthy households. A child from a mother who can decide on health, purchases, and visits to health institutions alone or jointly with the partner had 32% (aOR 0.68, 95% CI 0.56–0.84) lower odds of WaSt than their counterparts.

At the community-level factors, children from households with no access to newspaper, radio, or TV at least once per week had more than twice (aOR 2.55, 95% CI 1.78–3.65) increased odds of concurrent wasting and stung than their counterparts. Being from a rural residence, drinking water not on the premises, and defecating in the open field increase the odds of WaSt in model III but were not significantly associated in the adjusted model (Table 4).

**Table 4:** Effect of individual and community level factors on concurrent wasting and stunting among children 6–59 months in Ethiopia; data from EDHS 2000–2019.

		Model 1 (Empty model)	Model 2 Individual-level	Model 3 Community-level	Model 4 Combined final model
**Fixed effects model**					
**Individual-level variables**			aOR (95% CI)	aOR (95% CI)	aOR (95% CI)
Sex of a child	Boys		1.82 (1.50–2.21) **		1.81 (1.49–2.20) **
	Girls		Reference		Reference
Age of a child	6–11		Reference		Reference
	12–23		4.48 (3.16–6.35) **		4.56 (3.20–6.50) **
	24–35		3.59 (2.46–5.23) **		3.51 (2.39–5.15) **
	36–47		2.33 (1.58–3.44) **		2.44 (1.65–3.61) **
	48–59		1.77 (1.17–2.68)		1.85 (1.22–2.80) *
Preceding birth interval	Less than 2 years		1.79 (1.32–2.43) **		1.83 (1.34–2.49) **
	2 years		1.57 (1.20–2.06) **		1.59 (1.20–2.08) **
	3 years		1.20 (0.89–1.62)		1.19 (0.86–1.62)
	4 years and above		Reference		Reference
Birth size	Small/very small		2.53 (2.07–3.10) **		2.51 (2.05–3.08) **
	Average/large		Reference		Reference
Timely initiation of complementary foods	Yes		0.62 (0.48–0.78) *		0.62 (0.49–0.79) *
	No		Reference		Reference
Received vitamin A in the last 6 months	Yes		0.93 (0.76–1.14)		0.95 (0.77–1.16)
	No		Reference		Reference
Diarrhea in the last 2 weeks before the survey	Yes		1.53 (1.19–1.97) **		1.57 (1.22–2.03) **
	No		Reference		Reference
Cough in the last 2 weeks before the survey	Yes		0.92 (0.69–1.21)		0.93 (0.69–1.23)
	No		Reference		Reference
Fever symptoms in the last 2 weeks before the survey	Yes		1.61 (1.21–2.14) **		1.68 (1.25–2.20) **
	No		Reference		Reference
Anemia status	Severe		4.62 (2.87–7.42) **		4.70 (2.91–7.59) **
	Moderate		2.57 (2.01–3.27) **		2.51 (1.96–3.21) **
	Mild		1.90 (1.48–2.43) **		1.88 (1.47–2.42) **
	Not anemic		Reference		Reference
Maternal education	No formal education		1.77 (1.36–2.30) *		1.68 (1.28–2.19) **
	Have formal education		Reference		Reference
Maternal occupation	Housewife		1.16 (0.88–1.53)		1.13 (0.85–1.50)
	Agriculture		1.42 (1.04–1.95)		1.39 (1.01–1.92) **
	Business/ others		Reference		Reference
Wealth status	Poor		2.18 (1.67–2.85) **		1.98 (1.47–2.68) **
	Middle		1.85 (1.37–2.50) *		1.70 (1.23–2.33) *
	Rich		Reference		Reference
Women decide on health, purchases, and visits alone or jointly with their partners	No		Reference		Reference
	Yes		0.68 (0.56–0.83)		0.68 (0.56–0.84) **
**Community-level variables**					
Residence	Urban			Reference	Reference
	Rural			1.95 (1.49–2.55) *	0.68 (0.39–1.17)
Round trip time to get a water source	Water on premises			Reference	Reference
	30 min or less			1.52 (1.08–2.14) *	1. 34 (0.76–2.33)
	More than 30 min			1.55 (1.09–2.19) *	1.48 (0.84–2.59)
Toilet facility	Improved			Reference	Reference
	Unimproved			0.72 (0.58–0.91)	1.13 (0.74–1.72)
	Open defecation			1.73 (1.39–2.15) **	1.14 (0.74–1.75)
Media exposure: newspaper, radio, and TV at least once a week	Yes			Reference	Reference
	No			2.57 (2.04–3.25) **	2.55 (1.78–3.65) **
**Random effects**					
Community-level variance (SE)		0.679 (0.04)	1.478 (0.213)	0.670 (0.079)	1.489 (0.218)
ICC (%)		17.11	31.00	16.93	31.16
MOR		2.19	3.19	2.18	3.20
PCV (%)		Reference	−117.64	1.29	−119.29
**Model fit statistics**					
Log-likelihood		−5386.93	−1903.39	−5060.47	−1864.62
AIC		10777.85	3856.78	10136.93	3791.25
BIC		10793.38	4030.33	10198.76	4006.12

Significant at *p < 0.05; **p < 0.01.

### Random effects results

The empty model depicts that 17.11% of the variance in the odds of WaSt in Ethiopia could be attributed to variation between the clusters (ICC = 0.1711). The variation increased to 31% (ICC = 0.3100) in model-2 when only individual-level variables were fitted. The variation decreased to 16.93% (ICC = 0.1693) in model-3 with only community-level variables and finally increased to 31.16% (ICC = 0.3116) in model-4, which was adjusted for individual and community-level predictors. Model-4, the AIC and BIC values were lower than the previous three models, whereas the Log-likelihood ratio was the highest. Thus, model-4 is the best-fitted model to predict the likelihood of WaSt in Ethiopia. The median odds ratio (MOR) in the best-fitted model (model-4) showed that if a child moved from a cluster with a low risk of WaSt to a cluster with a high risk of WaSt, the median increase in the odds of WaSt would increase by three-fold (MOR = 3.20).

## Discussion

The prevalence of WaSt was 4.82% in Ethiopia. It was comparable with other studies in sub-Saharan Africa: 5% in Karamoja, Uganda (8), 6.2% in Niakhar, Senegal (4), less than 3.5% in Mozambique (5), and 5.8% in Kersa, Ethiopia (7). The trends of WaSt were declining from 7.69% in 2000 to 3.75% in 2019, with a percent change of −57.49% in Ethiopia. However, the percent change was slow from 2016 to 2019 (−1.5%). Based on the data from 2000 to 2019, the prevalence of WaSt, stunting, and wasting by 2030 is forecasted to be 0.28%, 22.4%, and 4.9%, respectively. Thus, with the current rate of reduction and the remaining 8 years (although the achievement is enormous), it’s impractical to end all forms of malnutrition by 2030.

In this study, boys were more likely to be concurrently wasted and stunted than girls. This is compatible with findings from Demographic and Health Surveys of Sub-Saharan Africa (33), a systematic review and meta-analysis (34), and other studies on wasting and stunting (11, 35–38). Some evidence in Ethiopia especially from rural communities shows that boys are preferred over girls and this is reflected in mothers’ health-seeking behavior and contraceptive utilization (39, 40). However, its effect on child-feeding behavior is unknown. Also, the exact cause linking child sex with malnutrition remains unclear. Biologically, hormonally, and immunologically boys are highly susceptible to undernutrition than girls. In terms of biology, boys have higher fat-free mass and a lower fat mass than girls which increases energy demands (4). Also, girls have thicker triceps and subscapular skinfolds than boys, while boys have a lower MUAC-for-age *z* score than girls, which makes boys more susceptible to undernutrition (4). Others associate gender-based variations with hormonal, immunological, and social factors (41, 42). For example, boys have low levels of growth hormones since the intrauterine life than girls which make them more susceptible to adverse events (43, 44). Similarly, hormones from the hypothalamic-pituitary-gonadal axis (testosterone, early disappearance of luteinizing hormone, and follicle-stimulating hormone) increase the susceptibility of boys to undernutrition than girls (4, 45, 46). However, gender-based variations were not reflected in nutrition policies and programs. Therefore, further studies are needed to understand the linkages and implications for nutrition policy that fill the gap related to sex variations.

WaSt decreases with rising age, suggesting that stunting is a persisting factor while wasting decreases with increasing age; the highest odd was at 12–23 months. This agrees with similar studies conducted elsewhere (4, 8). Since sub-optimal birth outcomes play a significant role in the development of WaSt, the timing of drivers of WaSt is important for preventive measures, including pre-birth and maternal health. A short preceding birth interval of less than or equal to 2 years was associated with WaSt in this study. Research findings showed that children with a short preceding birth interval had increased odds of multiple malnutrition (47), stunting (35, 47, 48), and wasting (49). The shorter birth interval may deplete maternal nutritional stores (both at macronutrient and micronutrient levels), which can negatively affect the health of the mother and child (47, 50). The recovery to the optimal status may be difficult if the mother is malnourished, breastfed the preceding child for an extended period, and carry out energy-demanding laborious work (51).On the other hand, the risk of WaSt increase with the competition between the preceding young child and succeeding infant for available resources, including breast milk, care, and attention from the mother/parents (52). Therefore, all stakeholders should encourage mothers to use modern contraception as early as possible to delay unintended pregnancy and have optimum child spacing.

In the current study, the odds of WaSt for small/very small children were twice higher than their average/larger counterparts. Small birth size may be associated with constrained intrauterine growth due to maternal, placental, fetal, genetic, or a combination of these factors (53). Besides, environmental factors such as smoking, indoor air pollution, and infections were linked with small birth sizes or low birth weight (54). Besides short-term complications, these children suffer from long-term health sequels, including abnormal physical growth and neurodevelopmental outcome (53, 54). Improvement of maternal nutrition and health during pregnancy that can be translated to adequate birth weight is vital to prevent intergenerational malnutrition (55). Antenatal visits are a massive window of opportunity to transmit health and nutrition messages and nutrition-based interventions.

Timely initiation of complementary foods (solid, semi-solid, and soft foods) in 6–8 months reduced the odds of WaSt by 29% in this study. Our study shows the proportion of children who started complementary foods within 6–8 months in Ethiopia was 74.56%. This indicates still one in four children had no access to complementary food. The household structure and composition can influence delayed initiation by influencing resources to the primary caregivers (56). As the child reaches 6 months, nutrient demands exceed what breast milk alone can satisfy. Therefore, the introduction of solid, semi-solid, and soft foods is required at this stage while continuing breastfeeding (57).

In this study, anemic children (severe, moderate, and mild) were at higher odds of WaSt than their non-anemic counterparts. The study also showed a high prevalence (51.17%) of anemia in children, where 28.17% were either moderate or severe anemia. According to the WHO classification (≥40% prevalence), anemia is a severe public health problem in Ethiopia among children (58). Children are at high risk for anemia due to high physiologic demands for growth and development, low iron stores, inadequate dietary intake of bioavailable iron, and iron depletion with frequent infections (59). Besides, poor socio-economic status, maternal anemia, birth spacing, multiple births, preterm and low birth weight, large family size, higher-order birth, rural residence, and low maternal literacy (60–64). Anemia results in growth retardation, impaired motor, and cognitive development, and increased morbidity and mortality (65, 66).

WaSt was associated with the mother’s lack of formal education in this study. This is compatible with a similar study conducted in Uganda (8). Maternal education affects child feeding and caring behavior (67). Similarly, maternal education broadens the knowledge base of optimal child feeding, dietary choices, seeking treatment from health institutions at times of illness, and better decision-making (25, 68–70). On the other hand, women’s age at first marriage, place of residence, and family’s wealth index were significant predictors of women’s education in Ethiopia (71). Thus, addressing the challenges of early marriage and establishing a system that improves their economic status such as access to financial resources, especially for rural uneducated mothers could influence child nutrition. Furthermore, as the mother’s education is an underlying determinant of malnutrition (72), promoting inclusive and equitable quality education by all girls is required according to the fourth sustainable development goal (73).

Children from a low/middle socio-economic status had higher odds of concurrent wasting and stunting compared to wealthy households. This is compatible with previous studies (74, 75). Mothers/parents with lower purchasing power may defer the initiation of complementary feeding, have inadequate access to high-quality and quantity of foods, increased exposure to health risks, compromised hygienic practices, and delay treatment-seeking from appropriate health institutions (76).

Media access to newspaper, radio, or TV at least once a week had 55% lower odds of concurrent wasting and stunting. In Ethiopia, most mothers had no formal education (71.04%) and lived in rural areas (87.33%). Exposure to mass media plays a vital role in reaching mothers with less education, rural residents, and hard-to-transfer health message in person (77). Media access increases mothers’/household members’ knowledge of WASH variables, optimal child feeding, child caring, and appropriate treatment seeking (78).

To the best of our knowledge, this is the first study to assess trends and predictors of WaSt at the national level in Ethiopia. Using multi-level analysis provides information about the efficacy of focusing intervention on clusters instead of individual-level variables only. This study also has a few limitations. First, as DHS data uses a cross-sectional study design, establishing a causal connection between the predictors and WaSt is impossible. Second, since the survey assessed the variables retrospectively, it may be prone to recall bias. Finally, the prevalence data (rather than the incidence rate) underestimate the real burden of wasting and WaSt in the community because a child can experience several acute episodes during a year (2, 79). Therefore, the true burden of WaSt may be much higher than the one reported in cross-sectional studies.

## Conclusion and recommendations

In this study, both individual and community-level factors were associated with concurrent wasting and stunting among children 6–59 months. Our analysis showed that the trends in WaSt are decreasing in Ethiopia. The study highlights the importance of considering individual and community-level factors to address WaSt, such as extra attention to boys and small size birth, empowering women to use family planning to prevent short birth intervals, and encouraging timely initiation of complementary feeding. Likewise, preventing diarrhea, fever, and anemia reduces the odds of WaSt. Furthermore, interventions are required to increase media access to health and nutrition information and improve girls’ education and income/wealth status.

## Data availability statement

The datasets presented in this study can be found in online repositories. The names of the repository/repositories and accession number(s) can be found in the article/supplementary material.

## Ethics statement

The studies involving humans were approved by National Research Ethics Review Committee. The studies were conducted in accordance with the local legislation and institutional requirements. Written informed consent for participation in this study was provided by the participants’ legal guardians/next of kin.

## Author contributions

AR conceptualized the study, obtained and analyzed the data, drafted the manuscript, interpreted the results and critically revised the manuscript. ÖB contributed to the conception of the idea, reviewed the manuscript and provided comments during the manuscript write-up. All authors contributed to the article and approved the submitted version.

## Conflict of interest

The authors declare that the research was conducted in the absence of any commercial or financial relationships that could be construed as a potential conflict of interest.

## Publisher’s note

All claims expressed in this article are solely those of the authors and do not necessarily represent those of their affiliated organizations, or those of the publisher, the editors and the reviewers. Any product that may be evaluated in this article, or claim that may be made by its manufacturer, is not guaranteed or endorsed by the publisher.

## Footnotes

^1^http://dhsprogram.com

## Abbreviations

AOR: Adjusted Odds Ratio
DHS: Demographic and Health Survey
EDHS: Ethiopia Demographic and Health Survey
ICC: Intra-Correlation coefficient
MOR: Median Odd Ratio
PCV: proportional change in variance
SE: Standard Error
SF: Supplementary File
SGD: Sustainable Development Goal
